# Species Extinction Curves Under Emergent Spatiotemporal Land‐Use Processes and the Role of Protected Areas

**DOI:** 10.1002/ece3.74433

**Published:** 2026-09-28

**Authors:** Nao Takashina, Buntarou Kusumoto

**Affiliations:** ^1^ Graduate School of Frontier Sciences The University of Tokyo Kashiwa Chiba Japan; ^2^ Faculty of Agriculture Kyushu University Kasuya Fukuoka Japan

**Keywords:** biodiversity conservation, endemics‐area relationship, extinction debt, protected areas, species‐area relationship

## Abstract

Anthropogenic land‐use change is a major driver of biodiversity loss. Predicting the resulting extinction risk is essential for balancing development and conservation, yet remains challenging due to the lack of frameworks that integrate dynamic human activity with species' spatial distributions. In this study, we develop a spatially explicit model that couples land‐use progression with species range‐size distributions to estimate species extinctions. This framework captures the stochastic emergence, spatial expansion, and merging of converted areas over time, while accounting for species' spatial range structure. We use it to compare extinction risk across land‐use dynamics, geographic gradients, and protected‐area implementation scenarios. Using endemic‐area relationships (EARs) to evaluate immediate species loss, we found that frequent emergence of new, spatially dispersed land‐use patches shifted species loss toward later stages of habitat conversion, producing a sharper increase than in scenarios assuming expansion from a single location. However, because multi‐site conversion also accelerates land conversion in time, this conversion pattern may pose a greater real‐time risk to biodiversity. We also investigate the role of protected areas, finding that earlier implementation can significantly reduce extinctions, although the effectiveness varies depending on land‐use patterns. These findings highlight the importance of incorporating spatiotemporal land‐use processes into extinction models and emphasize the urgency of timely conservation strategies to mitigate biodiversity loss.

## Graphical abstract

Dynamic land‐use change reshapes extinction risk by altering where and how converted areas emerge, expand, and merge. Our spatially explicit framework integrates these land‐use dynamics with species geographic ranges and protected‐area implementation. Frequent emergence of spatially dispersed land‐use patches shifts species loss toward later stages of habitat conversion but can accelerate land conversion in real time. Earlier establishment of protected areas reduces species loss, highlighting the importance of timely conservation under dynamic land‐use change.

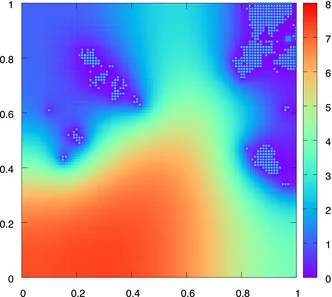

## 1 Introduction

Intensive land conversion on Earth has been altering ecosystems at an alarming rate, and subsequent species loss is one of the central issues for a sustainable society (Brennan et al. 2022; Millennium Ecosystem Assessment 2005; Pimm and Raven 2000; Watson et al. 2016; WWF 2022). Degradation of biodiversity causes loss of ecosystem services; hence, a reliable projection of extinction risk is essential to guide us in balancing development and conservation (IUCN 2022; Leclère et al. 2020; Millennium Ecosystem Assessment 2005). The establishment of protected areas (PAs) is also a key strategy for mitigating species loss caused by anthropogenic impacts (Leclère et al. 2020; Watson et al. 2014).

The extinction risk can be estimated based on the remaining geographic range size of a species (IUCN 2022). The species‐area relationship (SAR) and endemics‐area relationship (EAR), which quantify the number of all species and species endemic to a given region, respectively, have been extensively investigated in the literature on biogeography, community ecology, and macroecology, originally motivated by fundamental questions in ecology (MacArthur and Wilson 1963, 2001), and have become essential tools in conservation science (Matthews et al. 2021). They have been widely applied to estimate species extinction risk across scales ranging from local (He and Hubbell 2011) and regional‐continental (Hannah et al. 2020) to global (Gonçalves‐Souza et al. 2020; Malcolm et al. 2006), under drivers related to habitat loss (Gonçalves‐Souza et al. 2020; Rybicki and Hanski 2013) and climate change (Hannah et al. 2020; Malcolm et al. 2006).

While their broad applicability is well recognized, special attention, however, must be paid to the use of SAR and EAR in conservation practice. The shapes of the SAR and EAR depend on the spatial configurations of habitat loss (Kallimanis and Halley 2021), such as spatial patterns (Seabloom et al. 2002; Yin et al. 2021) and the direction of area loss (Keil et al. 2015). Also, habitat fragmentation has a considerable impact on species extinction estimates (Rybicki and Hanski 2013). Therefore, to evaluate the species extinction risk more realistically, it is essential to explicitly consider spatiotemporal land use dynamics, which describe the complex patterns of habitat loss and subsequent habitat fragmentation (Fahrig 2003). Existing models predicted land conversion patterns including deforestation patterns in the Brazilian Amazon using a high‐resolution land cover map combined with Bayesian hierarchical spatial models (Jaffé et al. 2021). Importantly, anthropogenic activities proceed simultaneously across regions (Long et al. 2021); a new land‐use event can take place at any point over time, accelerating habitat loss. Additionally, converted areas may merge when land‐use areas expand (Herold et al. 2003). Therefore, as land use progresses, the degree of habitat loss and fragmentation changes over time rather than being fixed. Despite this, many previous extinction estimates have relied on simplified land‐use representations, assuming single or unidirectional expansion patterns, or static fragmentation levels. Such approaches often did not take into account the ecological consequences of spatially dispersed and temporally dynamic land‐use processes. To address this gap, extinction risk will be evaluated within frameworks that explicitly incorporate the spatiotemporal complexity of land‐use progression. Such models provide a more realistic and nuanced understanding of biodiversity loss under ongoing anthropogenic pressures. Clearly, spatial distributions of species ranges also affect the shapes of the SAR and EAR, and consequently, the estimation of extinction risk using them (Matias et al. 2014; May et al. 2019). Therefore, in addition to modeling land‐use dynamics, it is essential to represent species ranges using ecologically grounded assumptions that capture key macroecological patterns across broad spatial scales, such as range size distributions and latitudinal gradients in species richness.

To meet these requirements, we developed a spatially explicit model to quantify the impact of land use on habitat loss under dynamic, simultaneous land‐use processes. The land‐use dynamics are formulated based on a Fisher–KPP‐type reaction–diffusion model (Logan 2007; Murray 2003), which represents the stochastic emergence, spatial spread, and merging of converted areas over time. The model flexibly accommodates the frequency of new land‐use emergence, the rate of expansion, density‐dependent effects on land‐use intensity, and the evaluation of policy interventions. Importantly, the model includes conventional area‐based extinction estimates as a limiting case: when no additional land‐use patches emerge, it reduces to a single‐origin expansion scenario analogous to the conventional sampling‐area setting. The ecological component of the model captures the spatial distribution of species ranges, based on a macroecological framework developed in (Takashina et al. 2019), allowing species distributions to reflect broad‐scale and global biogeographic patterns. In the analysis, we also consider scenarios both with and without large‐scale spatial gradients (e.g., latitudinal gradients of species diversity). We then use the model to examine how the timing of PA introduction influences conservation outcomes. This framework provides a means to assess extinction risk under dynamically changing patterns of habitat loss, and to systematically compare different land‐use and geographic scenarios.

Based on EAR‐based extinction estimates, our results showed that, compared with single‐origin land conversion, multiple, simultaneous land‐use events shifted species loss toward later stages of conversion but produced a sharper subsequent increase. This suggests that under more realistic land‐use dynamics, extinction risks may increase more abruptly than suggested by single‐origin land‐use scenarios. However, the introduction of PAs, even under such dynamic conditions, offers considerable benefits for biodiversity conservation by mitigating the accumulation of habitat loss and buffering species from extinction. These findings highlight the importance of incorporating spatiotemporal land‐use dynamics when assessing extinction risk, as well as the potential of timely conservation actions to bend the extinction curve.

## 2 Methods

We developed an integrated spatial model that couples species' geographic ranges with dynamic land‐use expansion at broad spatial scales.

The ecological component of the model builds on the macroecological framework developed by (Takashina et al. 2019), which describes species distributions based on geographic ranges and individual distributions within those ranges. Here, we focused on the geographic‐range level and refined this framework by generating range sizes that reproduce empirically observed, left‐skewed range‐size frequency distributions (RSFDs) on the log scale (Gaston 1998; Takashina et al. 2022), and by considering both random spatial placement and latitudinal gradients in species diversity.

Spatiotemporal land‐use dynamics is described by a Fisher–KPP formulation (Logan 2007; Murray 2003), which captures (i) expansion of land‐use patches under density‐independent or density‐dependent effects, (ii) the emergence of new land‐use patches, and (iii) the merging of expanding land‐use patches. Together, these processes produce dynamic changes in the degree of fragmentation over time.

By overlaying these ecological and socioeconomic components (Figure 1), we quantify species loss under multiple extinction definitions (complete or partial habitat loss) and evaluate how the timing of protected‐area implementation alters extinction outcomes. This framework grounded on previous studies (Takashina et al. 2019, 2022) enables consistent comparison of scenarios with and without latitudinal gradients and provides a general, spatially explicit baseline for assessing biodiversity responses to land‐use dynamics.

**FIGURE 1:**
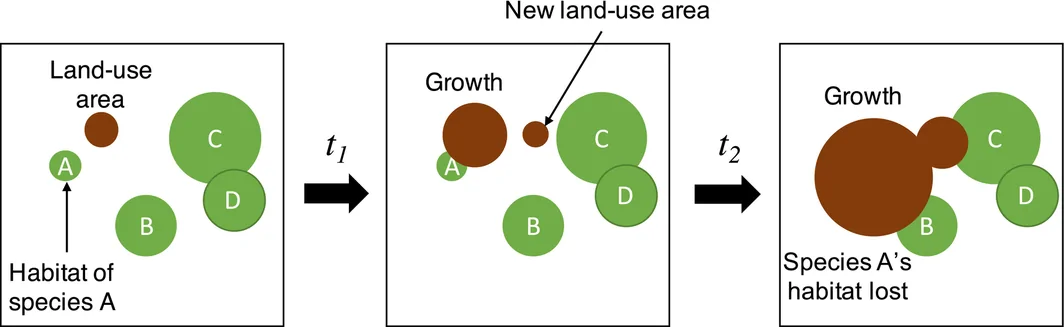
Schematic diagram of the developed model, in which land‐use dynamics and the geographic range of species are described in the model framework. Land‐use dynamics consist of the growth of land‐use (converted) area and the formation of new land‐use areas. As a result of the growth of land‐use areas, two separate land‐use areas may merge, which implies that the degree of fragmentation is not constant over time. Species loss is characterized by the complete/partial loss of the geographic range of a species.

### 2.1 Spatial Distribution of Species Ranges and Range‐Size Frequency Distributions

We represented species distributions at broad spatial scales using the macroecological framework developed by (Takashina et al. 2019), which is based on spatial point processes, that is, spatially explicit stochastic processes.

The species intensity, or expected species density, is denoted by $\lambda_{s}$. For a homogeneous spatial point process, the corresponding intensity measure in the focal region W is given by

(1) $$\mu_{s}\left(W\right)=\lambda_{s}\nu \left(W\right),$$

where $\nu \left(W\right)$ is the area of region W. The number of species in region W, denoted by $N\left(W\right)$, then follows a Poisson distribution with mean $\mu_{s}\left(W\right)$:

(2) $$P\left(N\right)=\frac{\mu_{s}{\left(W\right)}^{N}}{\text{N!}}e^{-\mu_{s}\left(W\right)}.$$

Each of the N species was then assigned a geographic range (Takashina et al. 2019).

Given the number of species and range sizes, each species' range was placed either randomly in space or arranged to generate a latitudinal gradient, that is, decreasing richness toward higher latitudes (Stevens 1989). Random placement was used because previous studies have shown that this range‐based framework can reproduce the well‐known triphasic species‐area relationship on a log–log scale when ranges are placed randomly and individuals are generated within each species' range (Grilli et al. 2012; Takashina et al. 2019), a pattern widely observed across taxa (Preston 1960; Rosenzweig 1995). Moreover, this framework can also reproduce well‐known relative species abundance patterns, such as Fisher's logseries and lognormal distributions (Takashina et al. 2019). Thus, despite its simplicity, random placement is theoretically grounded and provides a baseline spatial configuration for broad‐scale species distributions. The latitudinal‐gradient scenario can then be interpreted as an extension of random placement that incorporates a well‐established broad‐scale biogeographic pattern in species richness.

As part of this refinement, we specified species' range sizes so as to reproduce empirical RSFDs, another well‐known macroecological pattern (Brown et al. 1996; Gaston and He 2002). Range sizes vary greatly among species, and left‐skewed RSFDs on the log scale are widely observed across taxa (Blackburn and Gaston 1996; Brown et al. 1996; Takashina et al. 2022). For example, Gaston (1998) reported that the skewness of range‐size distributions ranged from −1.26 to −0.03 across taxa, including primates, birds, and beetles. We incorporated this empirical pattern of RSFDs into the following analysis. For simplicity, each species' range was represented as a circle, and its radius was drawn from a beta distribution with shape parameters α and β. We selected the beta distribution because its bounded support and flexible shape make it well suited for generating skewed distributions; after rescaling, it reproduces empirically observed left‐skewed range‐size frequency distributions on the log scale (Figure S.1).

Examples of species richness obtained by these processes are shown in Figure 2.

**FIGURE 2:**
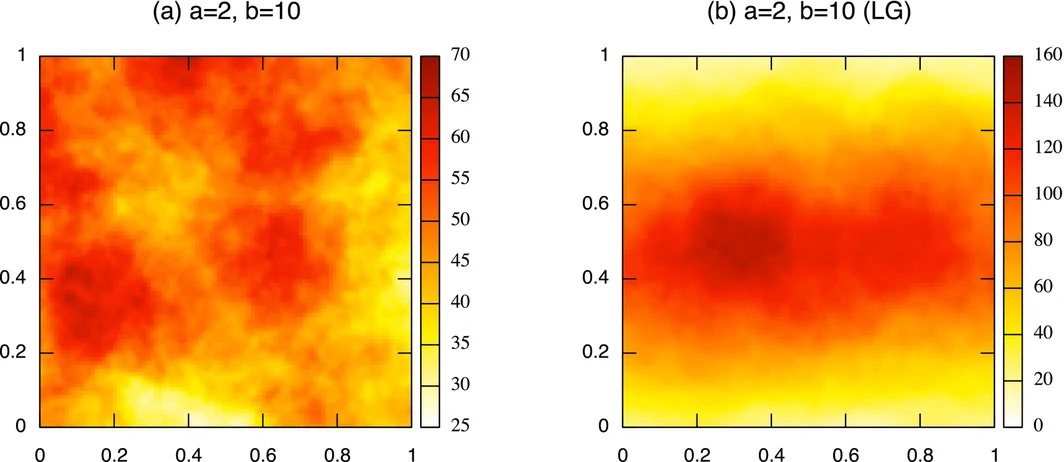
Examples of species richness (represented by colors) within the concerned domain in the scenario of (a) randomly placed range sizes (N=611) and (b) latitudinal gradient (LG) on y axis (N=799). Here, N denotes the total number of species in the domain, and α and β are the shape parameters of the beta distribution used to generate species range size frequency distributions.

### 2.2 Land‐Use Dynamics

Unlike existing area‐based extinction approaches, such as SAR‐ and EAR‐based estimates, which often rely on static or predefined spatial configurations of habitat loss (e.g., He and Hubbell 2011; Rybicki and Hanski 2013), real landscapes undergo multiple, spatially distributed land‐conversion processes. New converted areas continually emerge, and expanding patches can merge with others, producing a wide range of fragmentation levels over the course of land‐use conversion.

As the simplest possible model for extending existing area‐based approaches and capturing the spatiotemporal land‐use dynamics described above, we employed a Fisher–KPP‐type equation (Logan 2007; Murray 2003), which describes the local density‐dependent growth and spatial diffusion of land‐use intensity. The Fisher–KPP model is widely applied in ecology and various other fields, serving as a fundamental tool for modeling the growth and propagation of a concerned variable (Murray 2003). Importantly, this formulation includes conventional area‐based extinction estimates as a limiting case, in which a single converted region expands over time. Thus, our framework generalizes existing approaches by allowing multiple land‐use patches to emerge, expand, and merge dynamically. It also allows asymmetric land‐use expansion by permitting different diffusion coefficients along the *x*‐ and *y*‐axes, thereby relaxing the assumption of symmetric expansion often implicit in conventional settings. In our context, we denoted land‐use intensity by $u\left(x,t\right)$ at location $x=\left(x,y\right)$ and time t. The spatiotemporal land‐use dynamics are described by the following partial differential equation:

(3) $$u_{t}=\underset{\text{expansion}}{\underbrace{D_{x}u_{\mathrm{xx}}+D_{y}u_{\mathrm{yy}}}}+\underset{\text{production}}{\underbrace{\mathrm{ru}-\gamma u^{2}}}$$

where $D_{x}$ and $D_{y}$ are diffusion coefficients that determine the expansion speed of land‐use intensity along the x‐ and y‐axes, respectively. The parameters r and γ represent the intrinsic growth rate and the strength of density‐dependent saturation of land‐use intensity, respectively. The reaction term therefore describes logistic growth of land‐use intensity. This interpretation is consistent with urban‐growth studies that use logistic or sigmoid curves to describe city development as a process involving initial slow growth, accelerated fast growth, decelerated fast growth, and terminal slow growth (Chen 2023). More generally, in the context of land‐use dynamics, these terms can be interpreted as a phenomenological representation of increasing human inputs during development and their subsequent slowdown due to maintenance demands or resource constraints.

The dynamics were complemented by a no‐flux boundary condition, so that land‐use intensity did not flow across the boundary of the concerned region. The land‐use dynamics start with an initial input $u\left(x_{0},0\right)=r/\gamma$ where $x_{0}$ is drawn from a uniform distribution defined in the concerned region. At every time step, $t_{u}$, new land‐use occurs at a randomly selected point. We defined the land‐use of a location by the following indicator function:

(4) $$I\left(x\right)=\left\{\begin{array}{ll} 1, & u\left(x,t\right)>I\\ 0, & \text{otherwise} \end{array}\right.$$

where we define the region x to be converted (i.e., land use) if $I\left(x\right)=1$, and not converted otherwise. I indicates the threshold of land use intensity above which habitat conversion occurs. When I=0, the effects of density‐dependent interactions among different converted areas are ignored, and the patches expand independently. When I>0, the growth of land‐use intensity can be affected by density‐dependent processes, allowing density‐dependent interactions among patches during expansion. As a result, the total amount of land conversion can decrease, producing a clear difference from conventional scenarios in which converted area increases monotonically over time.

To investigate the effect of emergent land use, we introduced a parameter $t_{u}$, which determines the time interval at which new land‐use events are introduced into the system. Note that the conventional extinction estimate obtained from a single converted region is represented by the limit case $t_{u}=\infty$. In this scenario, only the initially converted area exists, and no additional land‐use patches appear over time; hence, the model reduces to a single‐origin conversion without emergent land use. When $t_{u}<\infty$, new land‐use patches emerge over time, allowing multiple converted areas to expand concurrently. These scenarios are summarized in Table 1.

**TABLE 1:** Classification of land‐use dynamics scenarios.

Condition	Scenario	Description
$t_{u}=\infty$	Single‐origin expansion	Conventional sample‐area setting with no emergent land‐use patches
$t_{u}<\infty ,I=0$	Multi‐origin expansion without density‐dependent effects	Multiple land‐use patches emerge and expand independently
$t_{u}<\infty ,I>0$	Multi‐origin expansion with density‐dependent effects	Multiple land‐use patches emerge and expand under density‐dependent effects

### 2.3 Species Extinction

Species extinction due to land conversion was estimated using the EAR, in which a species is considered extinct once its entire geographic range has been converted, resulting in immediate loss of the species (He and Hubbell 2011). To estimate extinction risk, we combined species' spatial range‐size distributions with land‐use dynamics. Specifically, spatial distributions of species ranges were first generated, followed by simulations of land‐use expansion. At each time step, we recorded the number of species whose entire geographic ranges had been converted. When land‐use patches expand independently (I=0), the converted area increases monotonically over time. In this case, the number of species lost at converted area A can be described as

(5) $$\mathrm{EAR}\left(A\right)=\text{number of species lost when the converted area is}\;A.$$

By contrast, when land‐use patches expand under density‐dependent effects (I>0), the converted area does not necessarily increase monotonically and can decrease over time. Under this situation, we defined the number of species lost at converted area A as the maximum value observed across all times at which the converted area reached A:

(6) $$\mathrm{EAR}\left(A\right)=\max_{A\left(t\right)=A}\mathrm{EAR}\left(A,t\right),$$

where $A\left(t\right)$ denotes the converted area at time t.

Rybicki and Hanski (2013), using simulations of metapopulation dynamics, argued that extinction risk may be underestimated by EAR when species only go extinct after a significant proportion of their habitat has been lost. In contrast, the SAR has been widely applied (Halley et al. 2013; He and Hubbell 2011) to species extinction estimates, though it can overestimate extinctions and contribute to the concept of extinction debt, which refers to a delayed extinction following habitat destruction (Tilman et al. 1994).

In light of these perspectives, we evaluated extinction using two criteria: species extinction following the loss of 90% or 95% of a species' geographic range, and extinction defined by SAR, where a species is considered extinct once any converted area overlaps with its geographic range. However, defining extinction based solely on specific habitat loss thresholds may itself introduce extinction debt. We discuss these extinction criteria further in the Discussion section.

### 2.4 Implementations of Protected Areas

We also examined the effect of PA implementation, where designated regions are made resistant to land conversion. As a simple representation of real‐world PA designation, we assumed that PAs could be introduced only in non‐converted areas. Thus, once the converted area exceeded predefined thresholds, PAs were assigned at once by selecting grids with the highest species richness among non‐converted areas, with their total extent specified in advance as a fixed proportion of all grids in the region. Figure 3 shows an example spatial realization of PA implementation, in which PAs covering 5% of all grids in the region were designated at once by selecting grids with the highest species richness among non‐converted areas after the converted area exceeded 20%.

**FIGURE 3:**
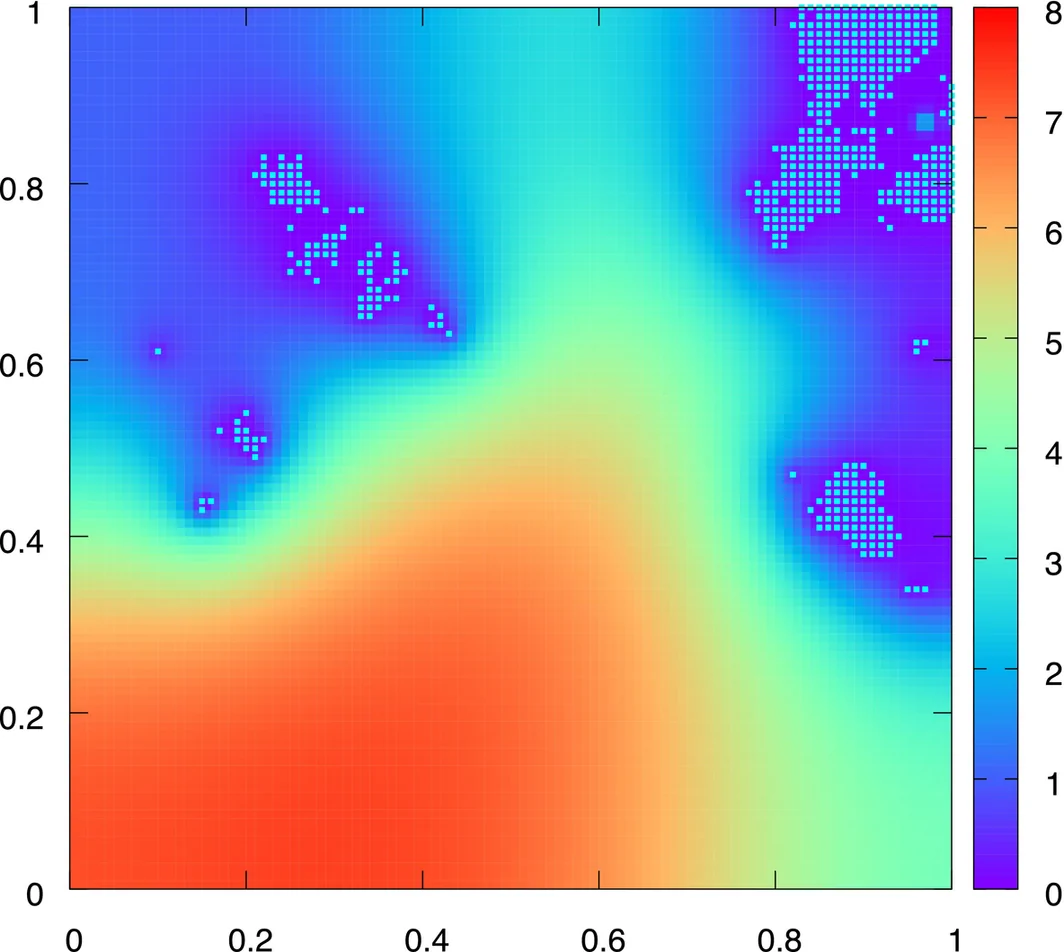
Example spatial realization of protected area implementation under dynamic land‐use expansion. The color scale represents land‐use intensity, and the light‐blue grid cells represent PAs. Once the converted area exceeded 20%, PAs covering 5% of all grids in the region were designated at once by selecting grids from the highest species‐richness patches among non‐converted grids. Once established, protected grids were represented as fixed internal cells and effectively acted as internal barriers or sinks that inhibited subsequent land‐use expansion. Parameter values in this illustrative example were α=2, β=10, $t_{u}=0.1$, and $D_{x}=D_{y}=0.005$.

In our spatially explicit model and numerical simulations, protected cells were represented as fixed internal cells where land‐use intensity was no longer updated and no new land‐use event could occur, thereby effectively acting as internal barriers or sinks that inhibited land‐use expansion. This representation allowed us to evaluate the buffering role of PAs under dynamic land‐use scenarios.

We further characterized the spatial arrangement of PAs using the pair correlation function $g\left(r\right)$, which measures the relative frequency of pairs of PA cells separated by distance r (Chiu et al. 2013; Illian et al. 2008). For a point pattern consisting of $N_{p}$ PA cells in the observation window W, we estimated $g\left(r\right)$ as

(7) $$\hat{g}\left(r\right)=\frac{\nu \left(W\right)}{\mathrm{\pi r}\Delta r\;N_{p}\left(N_{p}-1\right)}\sum_{i<j}I\left(r-\frac{\Delta r}{2}\leq ∥x_{i}-x_{j}∥<r+\frac{\Delta r}{2}\right)w_{\mathrm{ij}}^{-1},$$

where $\nu \left(W\right)$ is the area of focal region W, Δr is the distance‐bin width, $I\left(\cdot \right)$ is the indicator function, and $w_{\mathrm{ij}}$ is the translation edge‐correction factor (Illian et al. 2008). Values of $g\left(r\right)>1$ indicate that PA cells are more frequently separated by distance r than expected under random placement, whereas values of $g\left(r\right)<1$ indicate fewer pairs at that distance.

### 2.5 Numerical Simulations

By combining spatial distributions of species ranges and land‐use dynamics, we numerically investigated the risk of species extinction using various scenarios of spatial patterns of species ranges, land‐use dynamics, PA implementations, and species extinction.

For convenience, we use a scaled domain defined in the ranges 0≤x≤1 and 0≤y≤1. This did not affect our analysis because we were most interested in the relationship between the number of species that lost their range sizes and the proportional area of land uses. To avoid the edge effect in the numerical calculations, we consider an outer domain $W^{O}⊃W$ where the distance of each edge is $1+4\mu_{\mathrm{RS}}$, and $\mu_{\mathrm{RS}}$ is the mean diameter of a range‐size distribution ($\mu_{\mathrm{RS}}=a/\left(a+b\right)$; Figure S.2). Species ranges are placed within the domain $W^{O}$ and we include species in our numerical simulation if a species range overlaps with the concerned region W. Land use changes occur only within the concerned domain W. The domain W was discretized into 100×100 square grid cells with spatial intervals of Δx=Δy=0.01, and the resulting spatially discretized Fisher–KPP equation was integrated forward in time using the fourth‐order Runge–Kutta method with a time step of Δt=0.005.

Spatial distributions of species ranges are either the random placement or the placement to generate latitudinal gradients, under different range‐size distributions. The latitudinal gradient is incorporated into the generation of species ranges in space so that the location of the *y*‐axis (i.e., the north–south axis) follows a normal distribution, specifically, $N\left(\mu ,\sigma^{2}\right)$, where the mean and variance are set to μ=0.5 and $\sigma^{2}=0.04$. We also set the intensity measure (Equation 1) to $\mu_{s}\left(W\right)=10^{3}$ as an illustrative species‐pool size. This value is within a plausible order of magnitude for empirical species pools at regional to global spatial scales, such as those reported for reptiles (Cox et al. 2022), amphibians (LaFrance et al. 2024), and mammals (Burgin et al. 2025). This parameter can be adjusted based on species‐richness data for the taxonomic group and region of interest.

On the other hand, land‐use scenarios include homogeneous or inhomogeneous speeds of expansion of the converted areas ($D_{x}=D_{y}$ and $D_{x}\neq D_{y}$, respectively), with different rates of emergence of new land‐use areas $t_{u}$. Furthermore, we examine multiple extinction criteria, including SAR and EAR.

Lastly, we investigated how the implementation of PAs affected extinction estimates. Once the converted area reached a predetermined threshold (20% or 40%), we selected grids with the highest species richness from the non‐converted areas, corresponding to 5% of all grids in the region. Land‐use dynamics then continued, with these PAs remaining exempt from further conversion. In the calculation of the pair correlation function (Equation 7), we used translation edge correction. For the unit‐square observation window, the edge‐correction factor $w_{\mathrm{ij}}$ was given by

(8) $$w_{\mathrm{ij}}=\left(1-|dx_{\mathrm{ij}}|\right)\left(1-|dy_{\mathrm{ij}}|\right),$$

where $dx_{\mathrm{ij}}$ and $dy_{\mathrm{ij}}$ are the differences in the x‐ and y‐coordinates between PA cells i and j, respectively. The distance‐bin width was set to Δr=0.03.

For each scenario, we performed 100 simulations and showed the average extinction estimates obtained from different realizations of the spatial distribution of species ranges and land‐use dynamics. For the comparison across different values of $t_{u}$, we fixed the initial spatial distribution of species ranges within each replicate to ensure comparability. Key parameters and scenarios are summarized in Tables 2 and 3, respectively.

**TABLE 2:** Parameter values.

Symbol	Parameter	Value
r	Intrinsic growth rate of land‐use intensity	1
γ	Density‐dependent coefficient of land‐use indensity	0.1
$D_{x}$	Diffusion coefficient (west–east)	0.005
$D_{y}$	Diffusion coefficient (north–south)	0.0025, 0.005
$t_{u}$	Time interval controlling the emergence of new land‐use patches	0.05, 0.5, ∞
I	Threshold of land use intensity	0, 1
$\left(\alpha ,\beta \right)$	Beta distribution parameter	(2, 10), (2, 30), (10, 30)

**TABLE 3:** Simulation scenarios.

Scenario component	Values/description
Species‐loss estimate	SAR, EAR with 90%, 95%, or 100% converted range size defining extinction
Spatial distribution of species ranges	Random placement, latitudinal gradient
Protected area implementation	No PA, PAs introduced after 20% or 40% of the region had been converted

## 3 Results

### 3.1 Extinction Estimates Under Independent Land‐Use Expansion

Here, we examine extinction estimates when land‐use sites expand independently (I=0). Figure 4 shows endemics‐area relationships (upper panels), along with the five‐point moving average of the slope (lower panels). We found that the EAR curves tended to be convex, and the total number of species ranges lost was higher when the frequency of new land‐use events was lower (i.e., larger $t_{u}$). In other words, when a given amount of land is converted, fewer but larger patches of conversion result in a greater immediate loss of species ranges.

**FIGURE 4:**
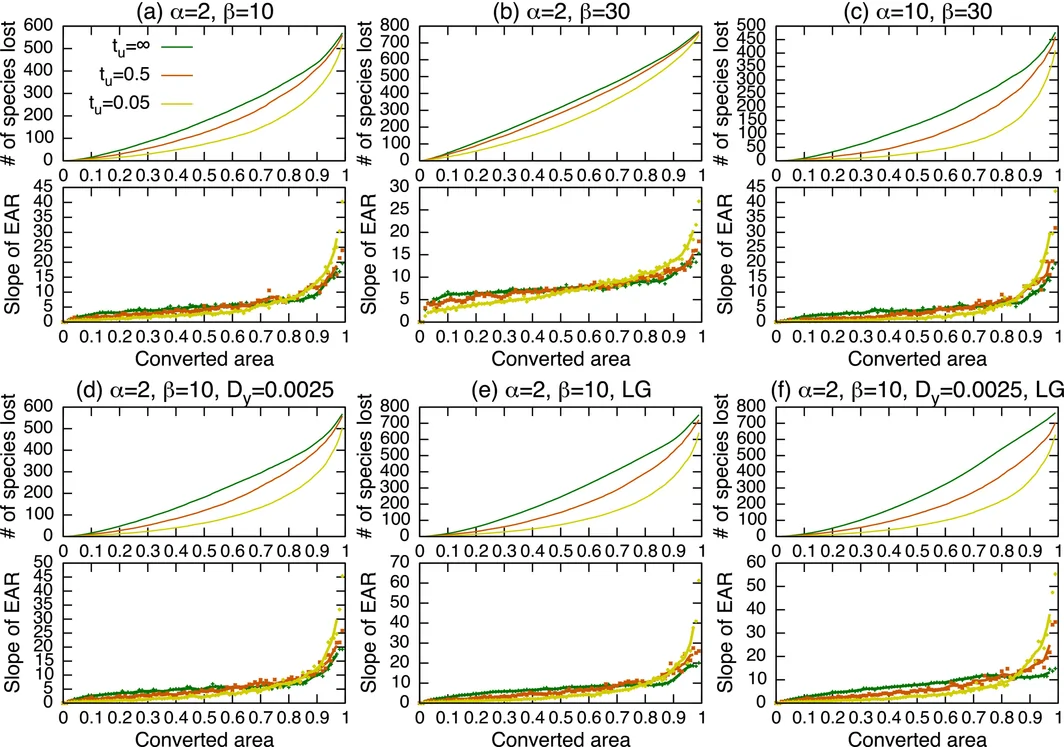
Endemics‐area relationships (EARs; top of each panel) and their slope (bottom of each panel) averaged over 100 simulation runs. In the bottom panels, points indicate the estimated slopes, and curves represent five‐point moving averages of these values. Within each panel and simulation run, the scenarios shared the same initial species distributions and richness.  Panels (a)–(f) represent different biological (characterized by the shape parameters for the beta distributions α and β, and the latitudinal gradient denoted by LG) and socioeconomic scenarios (characterized by the rate of land use expansion $D_{y}$). The parameter $t_{u}$ characterizes the frequency with which new land‐use occurs, and $t_{u}=\infty$ indicates that no additional land‐use area is formed. Other parameters used (if not specified) are $D_{x}=D_{y}=0.005$.

The slope of each EAR represents the change in the number of species lost per unit increase in converted area. In our simulations, the slope consistently increased as land use progressed, regardless of the frequency of new land‐use events. While the spatially contiguous EAR scenario ($t_{u}=\infty$), where converted land expands from a single location, resulted in greater species loss at earlier stages, the scenario with frequent, spatially dispersed land‐use events (smaller $t_{u}$) showed a slower initial increase in species loss but a much sharper rise at later stages of land‐use progression. This accelerating trend led to a steeper EAR slope in the later phase, indicating a higher incremental extinction risk under more realistic, simultaneous land‐use dynamics.

This upward pattern in the slope is widely observed in various scenarios especially when the frequency of occurrence of the new land use is high ($t_{u}$ is small), including scenarios where the speed of land use expansion is not symmetric in the north–south and east–west directions (i.e., $D_{x}\neq D_{y}$; Figure 4d), species ranges show a latitudinal gradient in space (Figure 4e), and these combinations (Figure 4f). In the asymmetric scenario, we set $D_{y}=0.0025$, corresponding to half of the baseline value $D_{x}=0.005$, but the qualitative EAR patterns were similar to those under symmetric diffusion. While these settings affected the trends of the EAR slope caused by a single urban area ($t_{u}=\infty$), qualitatively accelerating trends were observed in the slopes of the EARs for $t_{u}=0.5$ and 0.05.

As shown in Figure 5, land conversion proceeded faster when multiple land‐use patches expanded simultaneously at different locations (i.e., smaller $t_{u}$). Thus, in addition to the steeper increase in the EAR under small $t_{u}$, scenarios with more frequent emergence of new land‐use patches reached a given converted area in a shorter time. This indicates that species loss associated with land conversion can also proceed more rapidly in time under smaller $t_{u}$.

**FIGURE 5:**
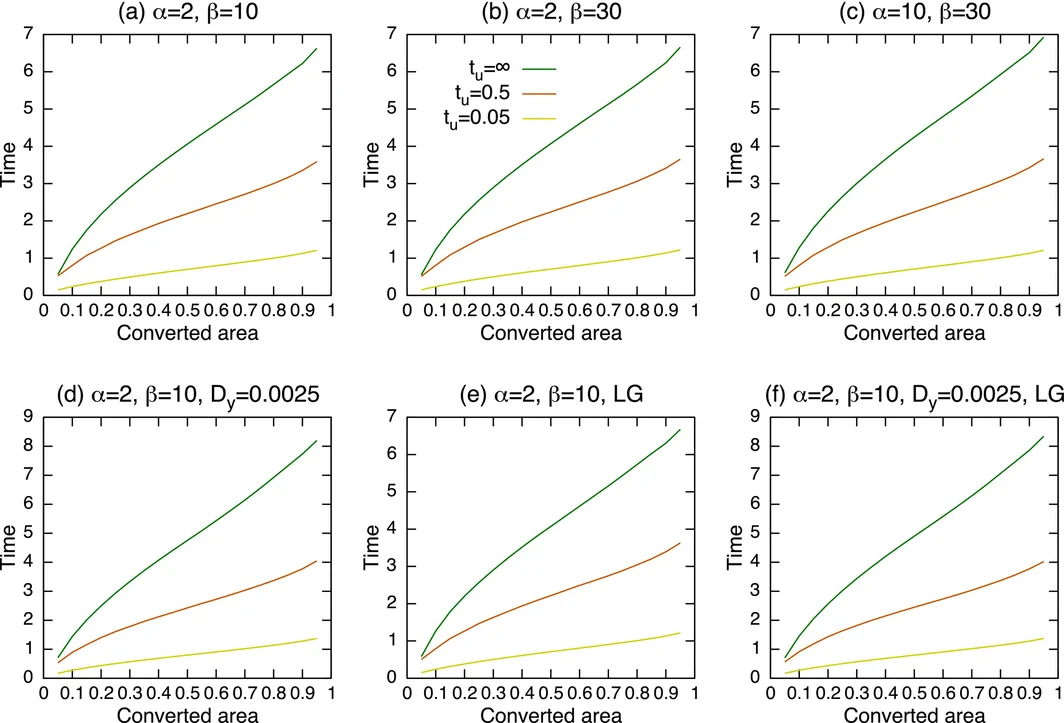
Relationship between the converted area and elapsed time. The values shown are the averages over 100 simulation runs. Within each panel and simulation run, the scenarios shared the same initial species distributions and richness. Panels (a)–(f) represent different biological (characterized by the shape parameters of the range size distributions α and β, and the latitudinal gradient denoted by LG) and socioeconomic (characterized by the rate of land‐use expansion $D_{y}$) scenarios. The parameter $t_{u}$ characterizes the frequency with which new land‐use occurs, and $t_{u}=\infty$ indicates that no additional land‐use area is formed. Other parameters used (if not specified) are $D_{x}=D_{y}=0.005$.

We also examined extinction estimates under two alternative criteria in which species were considered extinct after losing 95% (Figure 6) or 90% (Figure 7) of their geographic ranges, rather than only after complete range loss. Although similar extinction curves were obtained across criteria, some scenarios with latitudinal gradients showed peaks in the EAR slope at high levels of converted area, particularly in the bottom panels of Figures 6e,f and 7e,f. To contrast these EAR‐based results with the corresponding SAR‐based perspective, we also plotted backward SAR‐based estimates (Figure 8), which showed patterns opposite to those of the EARs.

**FIGURE 6:**
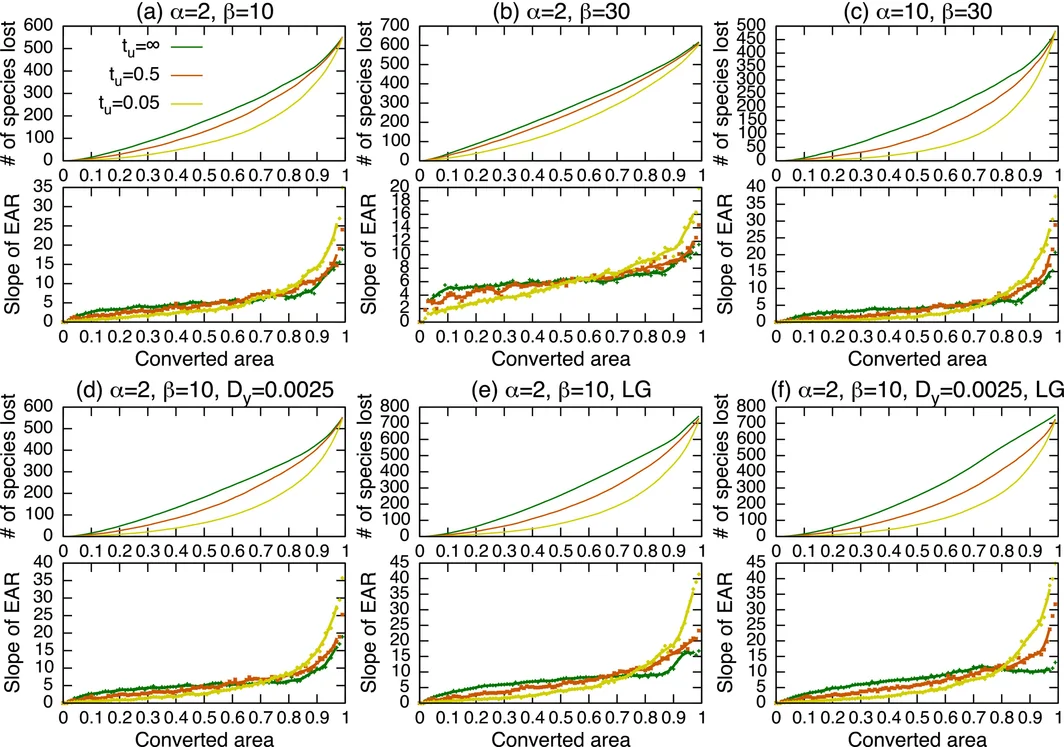
Endemics‐area relationships (EARs; top of each panel) and their slope (bottom of each panel) averaged over 100 simulation runs. In the bottom panels, points indicate the estimated slopes, and curves represent five‐point moving averages of these values. Within each panel and simulation run, the scenarios shared the same initial species distributions and richness. Extinction is defined as the range size when 95% of the area is converted. Panels (a)–(f) represent different biological (characterized by the shape parameters of the range size distributions α and β, and the latitudinal gradient denoted by LG) and socioeconomic (characterized by the rate of land use expansion $D_{y}$) scenarios. The parameter $t_{u}$ characterizes the frequency with which new land‐use occurs, and $t_{u}=\infty$ indicates that no additional land‐use area is formed. Other parameters used (if not specified) are $D_{x}=D_{y}=0.005$.

**FIGURE 7:**
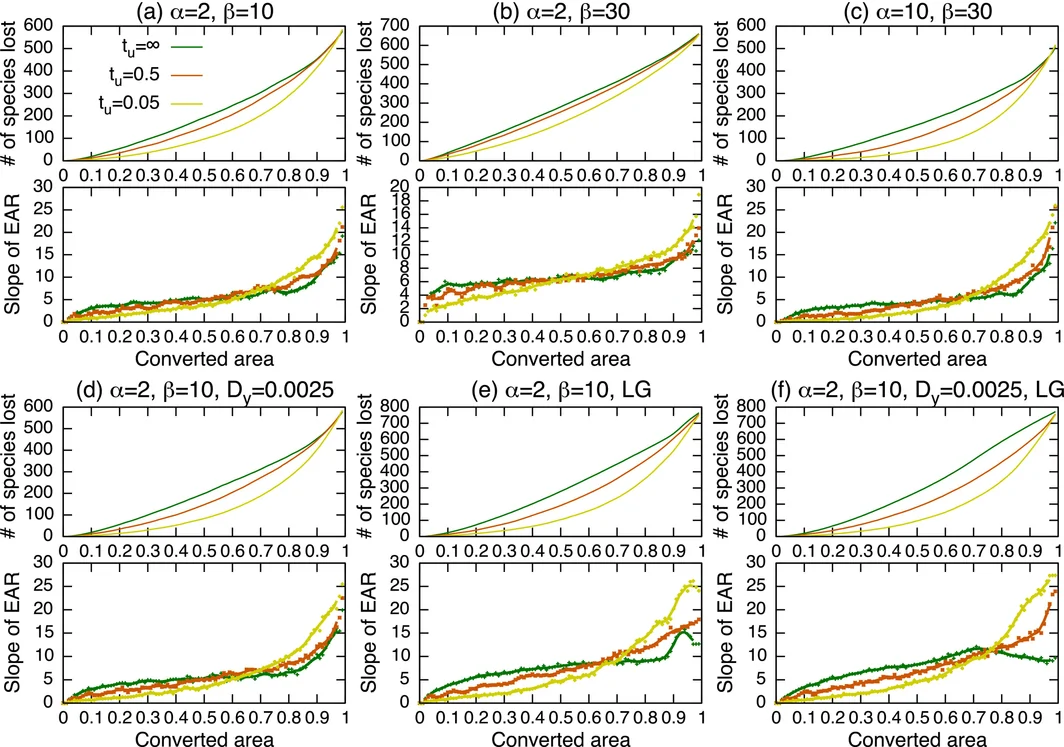
Endemics‐area relationships (EARs; top of each panel) and their slope (bottom of each panel) averaged over 100 simulation runs. In the bottom panels, points indicate the estimated slopes, and curves represent five‐point moving averages of these values. Within each panel and simulation run, the scenarios shared the same initial species distributions and richness. Extinction is defined as the range size when 90% of the area is converted. Panels (a)–(f) represent different biological (characterized by the shape parameters of the range size distributions α and β, and the latitudinal gradient denoted by LG) and socioeconomic (characterized by the rate of land use expansion $D_{y}$) scenarios. The parameter $t_{u}$ characterizes the frequency with which new land‐use occurs, and $t_{u}=\infty$ indicates that no additional land‐use area is formed. Other parameters used (if not specified) are $D_{x}=D_{y}=0.005$.

**FIGURE 8:**
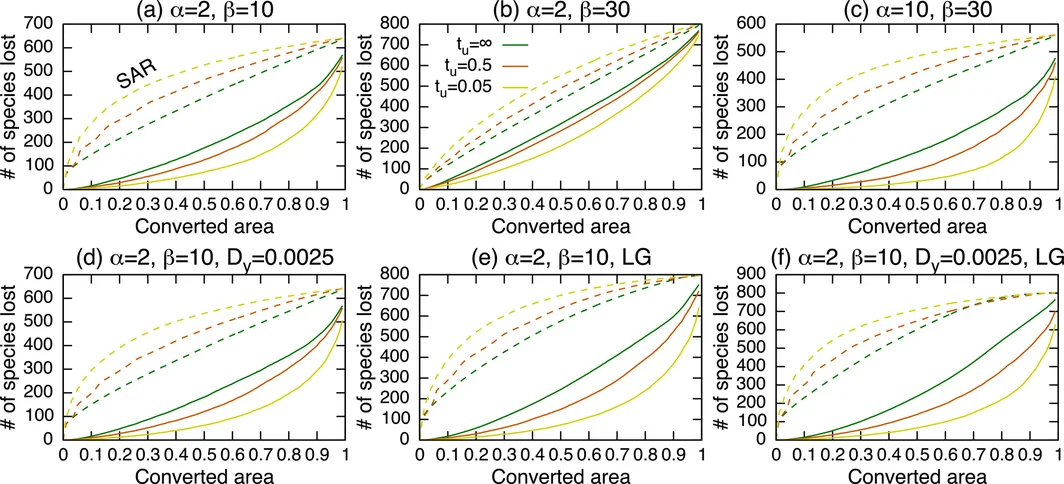
Endemics‐area relationships (EARs; solid curves) and species‐area relationships (SARs; dashed curves), averaged over 100 simulation runs. Within each panel and simulation run, the scenarios shared the same initial species distributions and richness. Panels (a)–(f) represent different biological (characterized by the shape parameters of the range size distributions α and β, and the latitudinal gradient denoted by LG) and socioeconomic (characterized by the rate of land use expansion $D_{y}$) scenarios. The parameter $t_{u}$ characterizes the frequency with which new‐land use occurs, and $t_{u}=\infty$ indicates that no additional land‐use area is formed. Other parameters used (if not specified) are $D_{x}=D_{y}=0.005$.

#### 3.1.1 Effects of Protected Areas Under Independent Land‐Use Expansion

Here, we investigated how the establishment of PAs affected EARs. PAs covering 5% of all grids in the region were designated at once in high‐richness grids among non‐converted areas after 20% or 40% of the region had been converted. We found that the PAs had a strong effect on bending the extinction curve (Figure 9 top panels) and reduce its slope (Figure 9 bottom panels). It offers the greater benefit if PAs are introduced earlier, even when the total PA extent is the same. However, patterns of land use also influence the effectiveness of PAs. In the spatially contiguous scenario ($t_{u}=\infty$), where land use spreads from a single location, species loss occurs rapidly in the early phase. Consequently, early implementation of PAs is more effective in bending the extinction curve (Figure 9a, top panel). In contrast, under scenarios with frequent, spatially dispersed land‐use events (e.g., $t_{u}=0.05$), species loss progresses more slowly, and the advantage of early PA implementation becomes less pronounced (Figure 9c, top panel).

**FIGURE 9:**
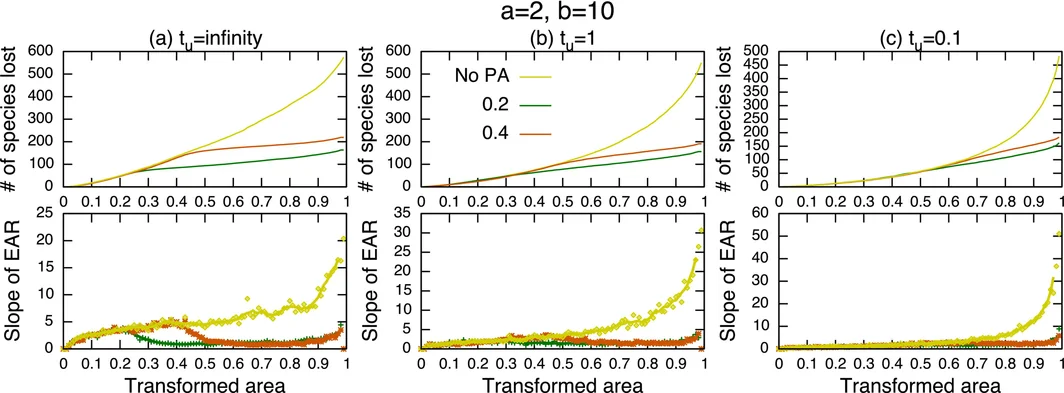
Effect of protected areas (PAs) on the endemics‐area relationships (EARs; top of each panel) and their slope (bottom of each panel) averaged over 100 simulation runs. In the bottom panels, points indicate the estimated slopes, and curves represent five‐point moving averages of these values. PAs were introduced only in non‐converted grids, with the number of selected grids set to 5% of all grids in the region, after the converted area exceeded 20% (green curve) or 40% (red curve). Within each panel and simulation run, the scenarios shared the same initial species distributions and richness. For comparison, we also plot the no‐PA scenario (yellow curve). The frequency of new land use to occur is (a) $t_{u}=\infty$ (i.e., single land use will spread the whole region); (b) $t_{u}=0.5$; and (c) $t_{u}=0.05$. Other parameters used are $D_{x}=D_{y}=0.005$.

Regarding the slope of the EAR, it declines after PA implementation and eventually converges to similar values between early and late PA introduction scenarios. This convergence was particularly notable when new land‐conversion patches emerged frequently, reducing the difference between early and late implementation (Figure 9c, bottom panel). Qualitatively similar results were observed in the other scenarios shown in Figures S.3–S.7, including different range‐size distributions, land‐use dynamics, and their combinations.

We further examined the spatial configuration of PAs using the pair correlation function $g\left(r\right)$ (Figure 10). In the spatially contiguous land‐use scenario ($t_{u}=\infty$), PAs were more strongly clustered at short distances, as indicated by larger values of $g\left(r\right)$, when they were introduced after 20% land conversion than when they were introduced after 40% land conversion. Qualitatively similar patterns were observed for the intermediate land‐use emergence scenario ($t_{u}=0.5$), although the differences in the pair correlation curves were less pronounced at short distances (r<0.15). By contrast, under frequent, spatially dispersed land‐use emergence ($t_{u}=0.05$), PAs were more strongly clustered when they were introduced earlier. These results indicate that realized PA configurations were strongly shaped by land‐use scenarios.

**FIGURE 10:**
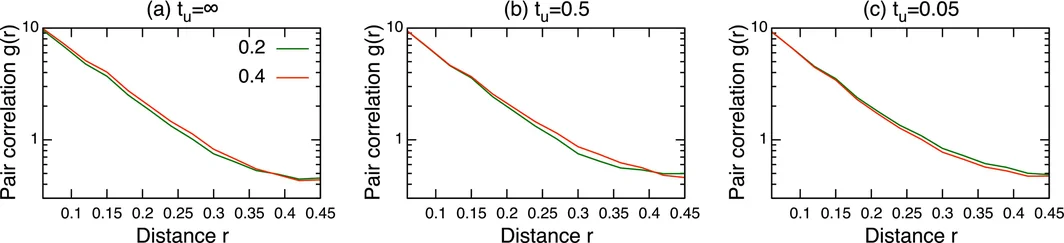
Pair correlation functions of PA cells corresponding to the PA implementation scenarios shown in Figure 9, averaged over 100 simulation runs. PAs were introduced after the converted area exceeded 20% (green curve) or 40% (red curve). The frequency of new land‐use emergence was controlled by $t_{u}$: (a) $t_{u}=\infty$, corresponding to single‐origin land‐use expansion with no additional land‐use emergence; (b) $t_{u}=0.5$; and (c) $t_{u}=0.05$. Other parameters were the same as in Figure 9.

### 3.2 Extinction Estimates Under Density‐Dependent Land‐Use Expansion

Here, we examine extinction estimates under density‐dependent land‐use expansion (I=1). Because density‐dependent effects among converted areas are not included in conventional extinction‐estimate frameworks, we did not conduct the same exhaustive set of analyses across extinction criteria and scenarios as in the density‐independent case. Instead, we focused on several basic scenarios to examine how density‐dependent land‐use expansion qualitatively alter extinction patterns.

Figure 11 shows the EARs under density‐dependent land‐use expansion, corresponding to the density‐independent results shown in Figure 4. Overall, the qualitative tendency was similar to that observed when I=0: more frequent emergence of new converted areas produced a slower initial increase in the EAR, followed by a sharper rise at higher levels of converted area. However, the differences among $t_{u}$ scenarios were smaller than those in Figure 4, particularly for the curves with $t_{u}=\infty$ and $t_{u}=0.5$.

**FIGURE 11:**
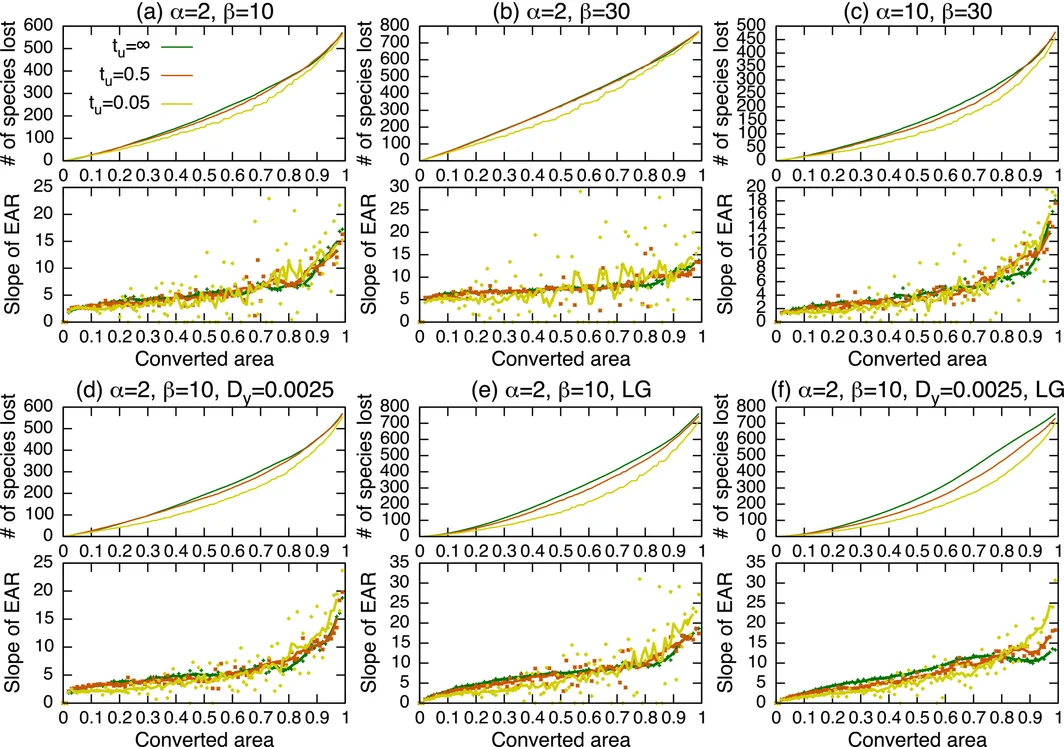
Endemics‐area relationships (EARs; top of each panel) and their slope (bottom of each panel) averaged over 100 simulation runs when the land‐use intensity threshold for conversion was set to I=1. In the bottom panels, points indicate the estimated slopes, and curves represent five‐point moving averages of these values. Within each panel and simulation run, the scenarios shared the same initial species distributions and richness. Panels (a)–(f) represent different biological (characterized by the shape parameters for the beta distributions α and β, and the latitudinal gradient denoted by LG) and socioeconomic scenarios (characterized by the rate of land use expansion $D_{y}$). The parameter $t_{u}$ characterizes the frequency with which new land‐use occurs, and $t_{u}=\infty$ indicates that no additional land‐use area is formed. Other parameters used (if not specified) are $D_{x}=D_{y}=0.005$.

A notable feature of the density‐dependent case was the larger variability among simulation runs, despite averaging over 100 replicates. This variability was especially evident in the slope of the EAR under the $t_{u}=0.05$ scenario. This pattern arises from the definition of the EAR in Equation (6): when converted areas exhibit density‐dependent effects, the proportion of converted area can increase and decrease over time, so the same converted‐area value can occur at multiple time points. Because smaller $t_{u}$ values generate a larger number of converted areas, the $t_{u}=0.05$ scenario was most strongly affected by this non‐monotonic behavior, leading to greater fluctuation in the estimated EAR slope.

Thus, density‐dependent land‐use expansion preserved the broad qualitative pattern observed in the density‐independent case, but reduced the separation among emergence‐frequency scenarios and introduced greater variability in EAR slopes when new converted areas emerged frequently.

#### 3.2.1 Effects of Protected Areas Under Density‐Dependent Land‐Use Expansion

We further examined how PA implementation affected EARs under density‐dependent land‐use expansion. As in the case without density dependence, we considered scenarios in which PAs covering 5% of all grids in the region were introduced in non‐converted areas after the converted area exceeded 20% or 40%. This allowed us to compare how the timing of PA implementation influenced extinction estimates when converted areas expand under density‐dependent effects.

Figure 12 corresponds to the PA scenario without density dependence shown in Figure 9. As in the scenario without PAs, the slopes of the EARs showed larger variability under density‐dependent land‐use expansion. Nevertheless, the qualitative pattern was broadly similar to that observed when I=0: PA implementation reduced species loss even when converted areas expand under density‐dependent effects, and earlier PA introduction provided greater conservation benefits than later introduction.

**FIGURE 12:**
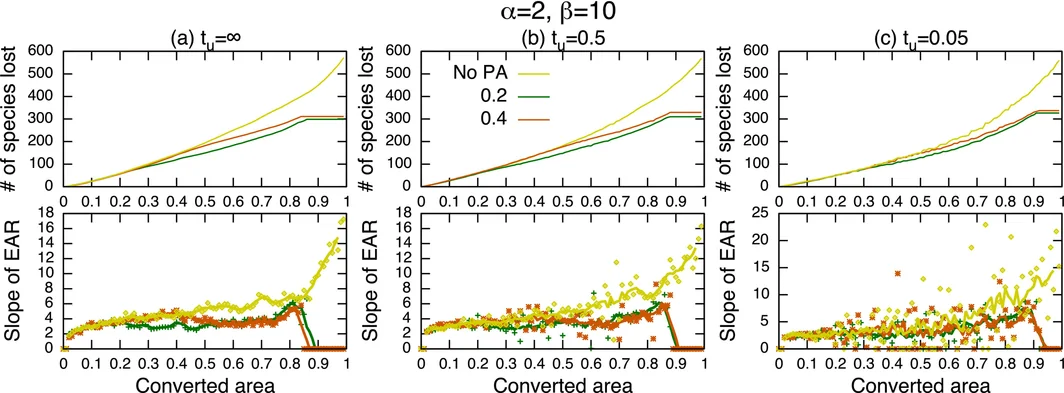
Effect of protected areas (PAs) on the endemics‐area relationships (EARs; top of each panel) and their slope (bottom of each panel) averaged over 100 simulation runs. In the bottom panels, points indicate the estimated slopes, and curves represent five‐point moving averages of these values. PAs were introduced only in non‐converted grids, with the number of selected grids set to 5% of all grids in the region, after the converted area exceeded 20% (green curve) or 40% (red curve). Within each panel and simulation run, the scenarios shared the same initial species distributions and richness. For comparison, we also plot the no‐PA scenario (yellow curve). The frequency of new land use to occur is (a) $t_{u}=\infty$ (i.e., single land use will spread the whole region); (b) $t_{u}=0.5$; and (c) $t_{u}=0.05$. Other parameters used are $D_{x}=D_{y}=0.005$.

Figure 13 shows the pair correlation functions of PA cells under density‐dependent land‐use expansion. Compared with the corresponding scenario without density dependence shown in Figure 10, the intersection between the pair correlation curves for PA introduction after 20% and 40% conversion shifted toward shorter distances under the $t_{u}=0.5$ scenario. In the $t_{u}=0.05$ scenario, such an intersection also emerged, indicating that density‐dependent effects among converted areas altered the spatial arrangement of realized PAs. Overall, however, the qualitative tendency in the relative magnitude of $g\left(r\right)$ among land‐use scenarios was similar to that observed when I=0.

**FIGURE 13:**
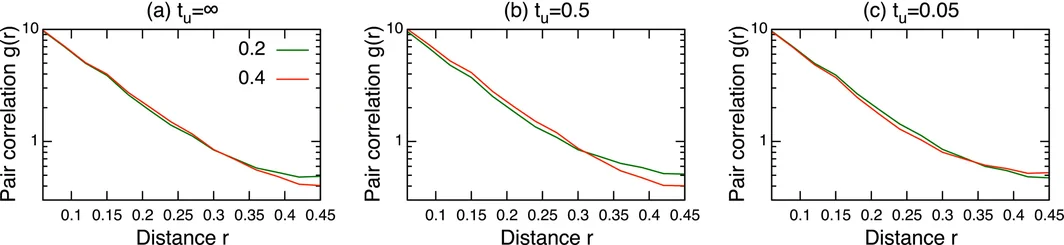
Pair correlation functions of PA cells corresponding to the PA implementation scenarios shown in Figure 12, averaged over 100 simulation runs. PAs were introduced after the converted area exceeded 20% (green curve) or 40% (red curve). The frequency of new land‐use emergence was controlled by $t_{u}$: (a) $t_{u}=\infty$, corresponding to single‐origin land‐use expansion with no additional land‐use emergence; (b) $t_{u}=0.5$; and (c) $t_{u}=0.05$. Other parameters were the same as in Figure 12.

Taken together, these results indicate that PAs continued to reduce extinction risk even when converted areas exhibit density dependence. However, the detailed spatial arrangement of PAs differed among land‐use scenarios, producing differences in the degree of PA clustering across distances.

## 4 Discussion

Projecting the impacts of land‐use change on biodiversity and species loss is essential for developing effective conservation strategies. Human activities create complex and dynamic mosaics of converted areas and remaining habitats (Bennett et al. 2006; Hanski 1998), yet extinction estimates have often relied on simplified or static representations of habitat loss. Here, we extended area‐based extinction estimates by coupling species' geographic ranges with spatiotemporal land‐use dynamics described by a Fisher–KPP framework. This allowed us to evaluate how the emergence, expansion, and merging of converted areas reshape species‐loss trajectories beyond the effect of total converted area alone.

Our results were consistent with previous studies showing that EARs increase with converted area (He and Hubbell 2011; Keil et al. 2015; Kinzig and Harte 2000), but they also revealed that the spatial dynamics of conversion strongly alter the shape of extinction curves. For the same total converted area, fewer but larger converted patches caused greater immediate species loss, whereas more fragmented, spatially dispersed conversion reduced early losses but produced a sharper increase at later stages. This pattern occurs because dispersed converted patches are less likely to fully overlap species ranges during early conversion, while complete conversion ultimately eliminates all species in the model. Thus, dynamic land‐use expansion can shift species loss toward later stages of habitat conversion, where extinction risk increases more abruptly.

However, area‐based species‐loss estimates do not explicitly include a temporal dimension, and their interpretation requires caution when land conversion occurs at multiple sites. Although multi‐site land conversion appears to delay species loss when evaluated against the proportion of converted area, it can substantially accelerate the actual pace of land conversion. When all conversion sites spread with the same diffusion coefficient within each scenario, the time required to reach a given proportion of converted area becomes shorter as new conversion events occur more frequently. Thus, the frequent emergence of new land‐conversion sites can make land conversion proceed faster than in the single‐origin scenario, indicating that an apparent delay in area‐based species loss does not necessarily imply a temporal delay in biodiversity loss.

Watson et al. (2016) reported that only 23.2% of terrestrial areas remain relatively wild. In the context of our results, this value suggests that many landscapes may already be approaching later stages of land conversion, where species loss can increase more abruptly. Thus, the current extent of human land‐use pressure, together with the continuing intensification of human activities, should be considered when evaluating the ecological consequences of anthropogenic land conversion (Kinzig and Harte 2000).

While the above prediction of EAR is based on immediate extinction that occurs when the entire habitat is converted (e.g., He and Hubbell 2011), other patterns can be obtained by setting alternative extinction criteria, such as extinction after 90% habitat conversion or SAR. The former can cause a peak of the slope in the extinction curve at a large converted area because a smaller area of land conversion will be sufficient to meet the extinction criteria compared to the criteria requiring complete destruction. In the limit as X approaches 0, the “X% range‐loss” criterion corresponds to the backward SAR‐based extinction estimate (He and Hubbell 2011), in which a species is counted as extinct once the converted area overlaps with its geographic range. He and Hubbell (2011) showed that EAR and backward SAR can form mirror‐image patterns under specific assumptions, such as random species distributions, and examined this relationship using 50‐ha forest plot data. We obtained qualitatively similar patterns in our simulations. However, no consensus exists on an adequate method for predicting extinction debt, which is still under active discussion (He and Hubbell 2011; Ridding et al. 2021; Rybicki and Hanski 2013).

Except for the immediate extinction criteria (i.e., EAR), the above‐mentioned extinction criteria induce extinction debt, which is another source of uncertainty in the prediction of species loss. Figueiredo et al. (2019) reported that extinction debt can span from a few years to 570 years, illustrating the large uncertainty involved in translating area‐based extinction estimates into temporal predictions. In addition, the duration of the extinction debt can be rather long when the species are close to their extinction thresholds (Hanski and Ovaskainen 2002). The magnitude of the extinction debt may depend on the species (Hylander and Ehrlén 2013), and therefore, non‐immediate extinction criteria inherently vary between species. Hence, one must bear multitudinous uncertainties in the time window if one uses the non‐immediate extinction criteria.

Our simulation demonstrated the strong effect of PAs on bending the extinction curve. It was also shown that the earlier introduction of PAs can save a greater number of species. However, notably, our discussion is based on immediate extinction, and the possibility of causing an extinction debt remains. In fact, if one uses alternative extinction criteria, such as 90% habitat destruction, a small fraction of PAs will not play a crucial role in bending the species extinction curve in the long run. That is, it may be more accurate to say that PAs can save numerous species from immediate extinction in the short term; however, sufficiently large PAs are needed to suppress the extinction debt in the long term. Moreover, previous studies have identified the issue of missed conservation opportunities arising from delays in establishing PAs due to the risk of habitat loss (Grantham et al. 2009). Our study provided theoretical evidence that despite the preservation of a specific habitat area, delays in the implementation of PAs can expedite the threat of extinction through foregone conservation opportunities. This highlights the importance of the prompt implementation of area‐based conservation measures, as stated in the Global Biodiversity Framework (Convention on Biological Diversity 2022).

While we focused on endemic species in a pristine environment, rich biodiversity often forms in socio‐ecological production landscapes where anthropogenic activity is maintained, such as traditional agricultural landscapes (Katoh et al. 2009). In addition, other effective area‐based conservation measures (OECMs) have been recognized for their complementary importance to PAs, and are beginning to be integrated into area‐based conservation measures (Maxwell et al. 2020). Novel ecosystems incorporating moderate human inputs provide considerable ecosystem services to humanity and are the foundation for resilient socioecological systems (Fukamachi 2020). Integrating these perspectives requires consideration of more general scenarios in which converted areas exhibits density‐dependent effects (I>0) and PAs do not completely exclude land use. Further discussion of land‐use types and diverse biodiversity conservation concepts is necessary to deepen our understanding of pathways toward a sustainable society.

## 5 Conclusions

We examined species extinction curves by incorporating dynamic land‐use processes into an area‐based extinction framework. A consistent accelerating trend in species loss was observed under the immediate extinction scenario defined by the EAR, which did not consider the effects of PAs. When alternative extinction definitions were applied, different patterns emerged, highlighting the sensitivity of extinction projections to underlying assumptions. These findings underscore the large uncertainties associated with extinction timing and debt, and emphasize the need for further research to establish consistent, ecologically grounded extinction criteria in the context of habitat destruction.

Our results also underscore the substantial role that PAs can play in bending extinction curves. In particular, we found that early implementation of PAs offers pronounced benefits in mitigating immediate species loss. Recent studies have similarly emphasized the conservation value of small but strategically placed habitats that deliver substantial ecological benefits and services (Riva et al. 2022; Valdés et al. 2020). Protecting such areas through timely interventions is essential, particularly through collective and community‐driven conservation efforts (Riva et al. 2022).

To address the limited integration of dynamic land‐use processes into area‐based extinction estimates, we developed a spatially explicit model that captures key features of landscape transformation, including the emergence, expansion, and merging of converted areas. While this framework provides a foundation for analyzing extinction under changing land use, it remains a simplified representation. Future improvements should include the incorporation of diverse land‐use types (e.g., agriculture, urbanization) with varying ecological impacts and expansion dynamics. Such refinements, along with clearer definitions of extinction thresholds, will enhance our ability to understand and manage the complex interplay between anthropogenic pressures and biodiversity loss.

## Author Contributions

**Nao Takashina:** conceptualization (lead), formal analysis (lead), funding acquisition (lead), investigation (lead), methodology (lead), validation (equal), visualization (lead), writing – original draft (equal), writing – review and editing (equal). **Buntarou Kusumoto:** validation (equal), writing – original draft (equal), writing – review and editing (equal).

## Funding

N.T. was financially supported by Japan Society for the Promotion of Science (JSPS) KAKENHI (grant number 23K28275).

## Conflicts of Interest

The authors declare no conflicts of interest.

## Supporting information


**Figure S.1:** Realized range size frequency distributions with an expected number of species $10^{5}$ for different shape parameters of the beta distribution α and β. LG indicates the effect of latitudinal gradients in the spatial distribution of species ranges. The skewness of each range‐size distribution is (a) −0.737, (b) −0.833, (c) −0.548, and (d) −1.247.
**Figure S.2:** Representation of the concerned domain W and its outer space $W^{O}$. The concerned domain is defined in the range 0≤x≤1 and 0≤y≤1, and the outer domain is defined in the range $-2\mu_{\mathrm{RS}}\leq x\leq 1+2\mu_{\mathrm{RS}}$ and $-2\mu_{\mathrm{RS}}\leq y\leq 1+2\mu_{\mathrm{RS}}$ where $\mu_{\mathrm{RS}}$ is the mean of a range‐size distribution defined by the parameters of the beta distribution $\mu_{\mathrm{RS}}=\alpha /\left(\alpha +\beta \right)$.
**Figure S.3:** Effect of protected areas (PAs) on the endemics‐area relationships (EARs; top of each panel) and their slope (bottom of each panel) averaged over 100 simulation runs. In the bottom panels, points indicate the estimated slopes, and curves represent five‐point moving averages of these values. PAs introduced 5% in the whole region after the converted area exceeds 20% (green curve) or 40% (red curve). For the comparison, we also plot the no‐PA scenario (yellow curve). Within each panel and simulation run, the scenarios shared the same initial species distributions and richness. The parameter $t_{u}$ characterizes the frequency with which new land‐use occurs, and $t_{u}=\infty$ indicates that no additional land‐use area is formed. Other parameters used (if not specified) are $D_{x}=D_{y}=0.005$.
**Figure S.4:** Effect of protected areas (PAs) on the endemics‐area relationships (EARs; top of each panel) and their slope (bottom of each panel) averaged over 100 simulation runs. In the bottom panels, points indicate the estimated slopes, and curves represent five‐point moving averages of these values. Protected areas (PAs) introduced 5% in the whole region after the converted area exceeds 20% (green curve) or 40% (red curve). For the comparison, we also plot the no‐PAs scenario (yellow curve). Within each panel and simulation run, the scenarios shared the same initial species distributions and richness. The parameter $t_{u}$ characterizes the frequency with which new land‐use occurs, and $t_{u}=\infty$ indicates that no additional land‐use area is formed. Other parameters used (if not specified) are $D_{x}=D_{y}=0.005$.
**Figure S.5:** Effect of protected areas (PAs) on the endemics‐area relationships (EARs; top of each panel) and their slope (bottom of each panel) averaged over 100 simulation runs. In the bottom panels, points indicate the estimated slopes, and curves represent five‐point moving averages of these values. Non‐symmetric rate of land‐use expansion was assumed. PAs introduced 5% in the whole region after the converted area exceeds 20% (green curve) or 40% (red curve). For the comparison, we also plot the no‐PA scenario (yellow curve). Within each panel and simulation run, the scenarios shared the same initial species distributions and richness. The parameter $t_{u}$ characterizes the frequency with which new land‐use occurs, and $t_{u}=\infty$ indicates that no additional land‐use area is formed.
**Figure S.6:** Effect of protected areas (PAs) on the endemics‐area relationships (EARs; top of each panel) and their slope (bottom of each panel) averaged over 100 simulation runs. In the bottom panels, points indicate the estimated slopes, and curves represent five‐point moving averages of these values. Latitudinal gradient (LG) was assumed. PAs introduced 5% in the whole region after the converted area exceeds 20% (green curve) or 40% (red curve). For the comparison, we also plot the no‐PA scenario (yellow curve). Within each panel and simulation run, the scenarios shared the same initial species distributions and richness. The parameter $t_{u}$ characterizes the frequency with which new land‐use occurs, and $t_{u}=\infty$ indicates that no additional land‐use area is formed. Other parameters used (if not specified) are $D_{x}=D_{y}=0.005$.
**Figure S.7:** Effect of protected areas (PAs) on the endemics‐area relationships (EARs; top of each panel) and their slope (bottom of each panel) averaged over 100 simulation runs. In the bottom panels, points indicate the estimated slopes, and curves represent five‐point moving averages of these values. Latitudinal gradient (LG) and non‐symmetric rate of land‐use expansion ($D_{y}=0.0025$) were assumed. PAs introduced 5% in the whole region after the converted area exceeds 20% (green curve) or 40% (red curve). The values shown are the averages over 100 simulation runs. For the comparison, we also plot the no‐PA scenario (yellow curve). Within each panel and simulation run, the scenarios shared the same initial species distributions and richness. The parameter $t_{u}$ characterizes the frequency with which new land‐use occurs, and $t_{u}=\infty$ indicates that no additional land‐use area is formed.

## Data Availability

We provide the baseline C code, example plotting scripts, and user instructions for the spatially explicit land‐use model under open access at https://doi.org/10.5281/zenodo.21990283.
