# Supplementary material for: Species Extinction Curves Under Emergent Spatiotemporal Land‐Use Processes and the Role of Protected Areas
Source: Ecol Evol. 2026 Sep 28;16(10):e74433. doi: 10.1002/ece3.74433 (PMC13620222; doi:10.1002/ece3.74433)
Supplement: Supplementary file 1 [file ECE3-16-e74433-s001.pdf]

Supporting Information

Species extinction curves under emergent spatiotemporal land-use processes and the role of protected areas

Nao Takashina and Buntarou Kusumoto

A Appendix Figures

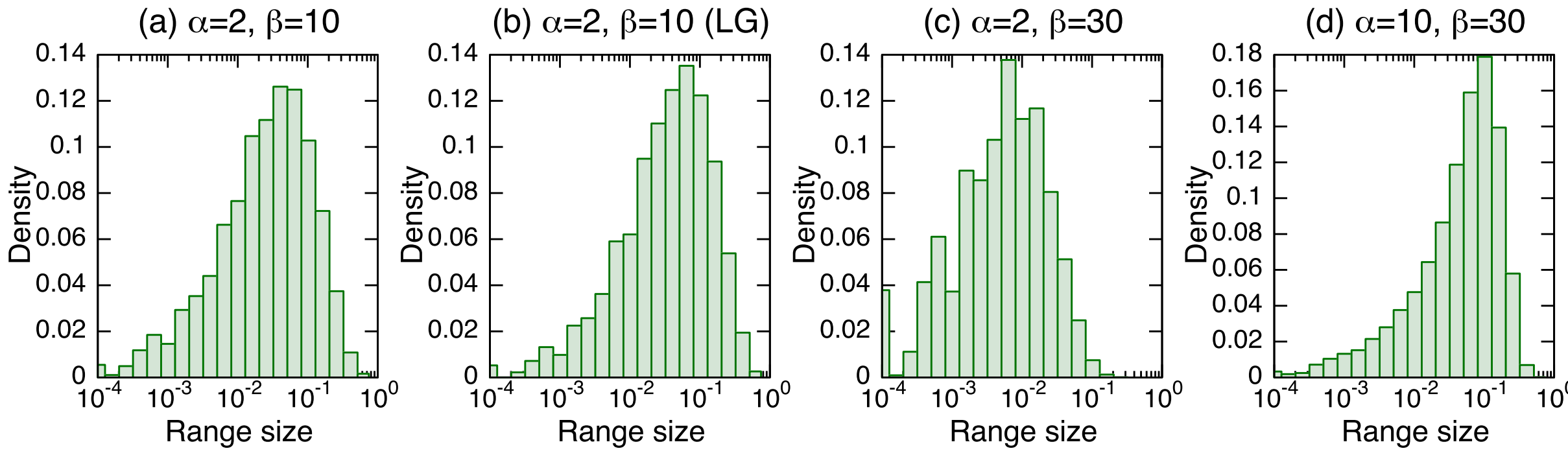

Figure S.1: Realized range size frequency distributions with an expected number of species 10^5 for different shape parameters of the beta distribution α and β. LG indicates the effect of latitudinal gradients in the spatial distribution of species ranges. The skewness of each range-size distribution is (a) -0.737, (b) -0.833, (c) -0.548, and (d) -1.247.

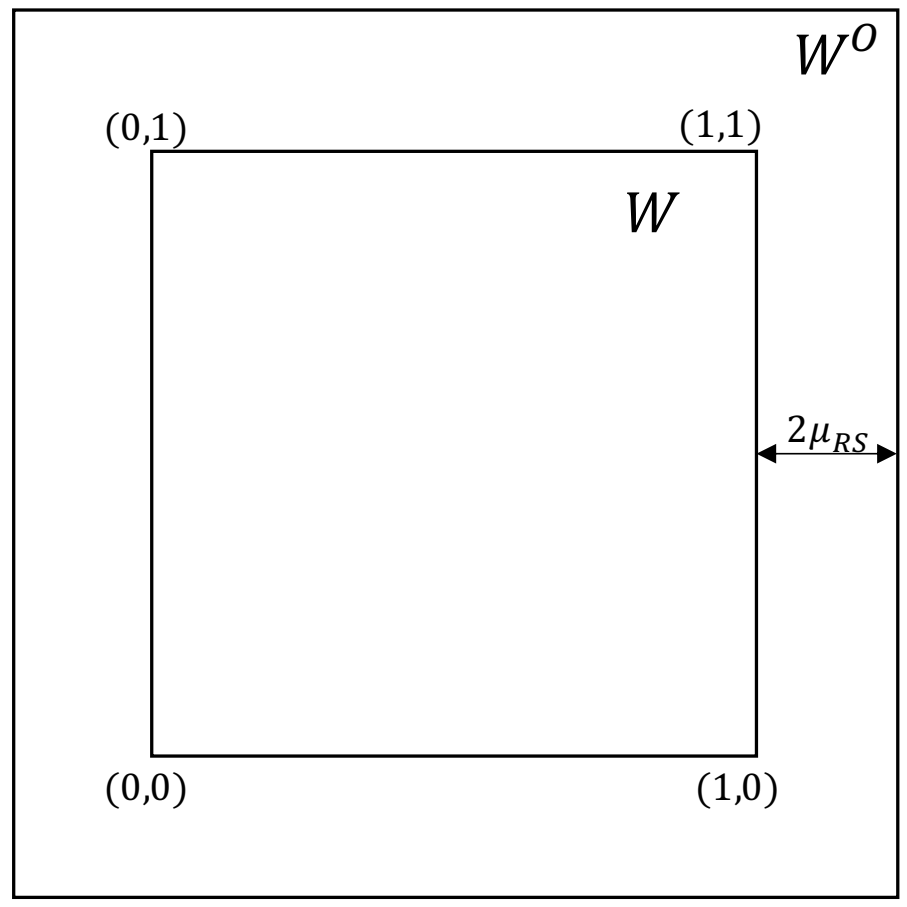

Figure S.2: Representation of the concerned domain W and its outer space W^O. The concerned domain is defined in the range $0 \leq x \leq 1$ and $0 \leq y \leq 1$, and the outer domain is defined in the range $-2\mu_{RS} \leq x \leq 1 + 2\mu_{RS}$ and $-2\mu_{RS} \leq y \leq 1 + 2\mu_{RS}$ where μ_{RS} is the mean of a range-size distribution defined by the parameters of the beta distribution $\mu_{RS} = \alpha/(\alpha + \beta)$.

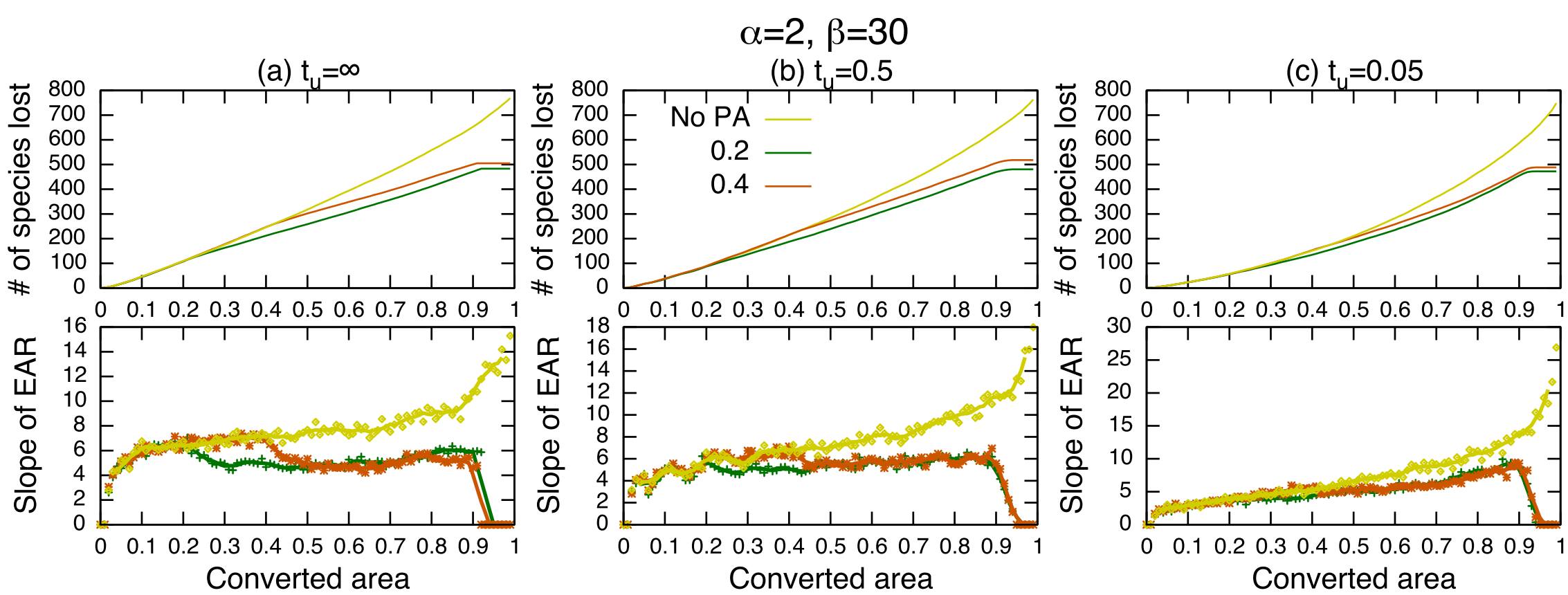

Figure S.3: Effect of protected areas (PAs) on the endemics–area relationships (EARs; top of each panel) and their slope (bottom of each panel) averaged over 100 simulation runs. In the bottom panels, points indicate the estimated slopes, and curves represent five-point moving averages of these values. Protected areas (PAs) introduced 5% in the whole region after the converted area exceeds 20% (green curve) or 40% (red curve). For the comparison, we also plot the no-PA scenario (yellow curve). Within each panel and simulation run, the scenarios shared the same initial species distributions and richness. The parameter t_u characterizes the frequency with which new land-use occurs, and $t_u = \infty$ indicates that no additional land-use area is formed. Other parameters used (if not specified) are $D_x = D_y = 0.005$.

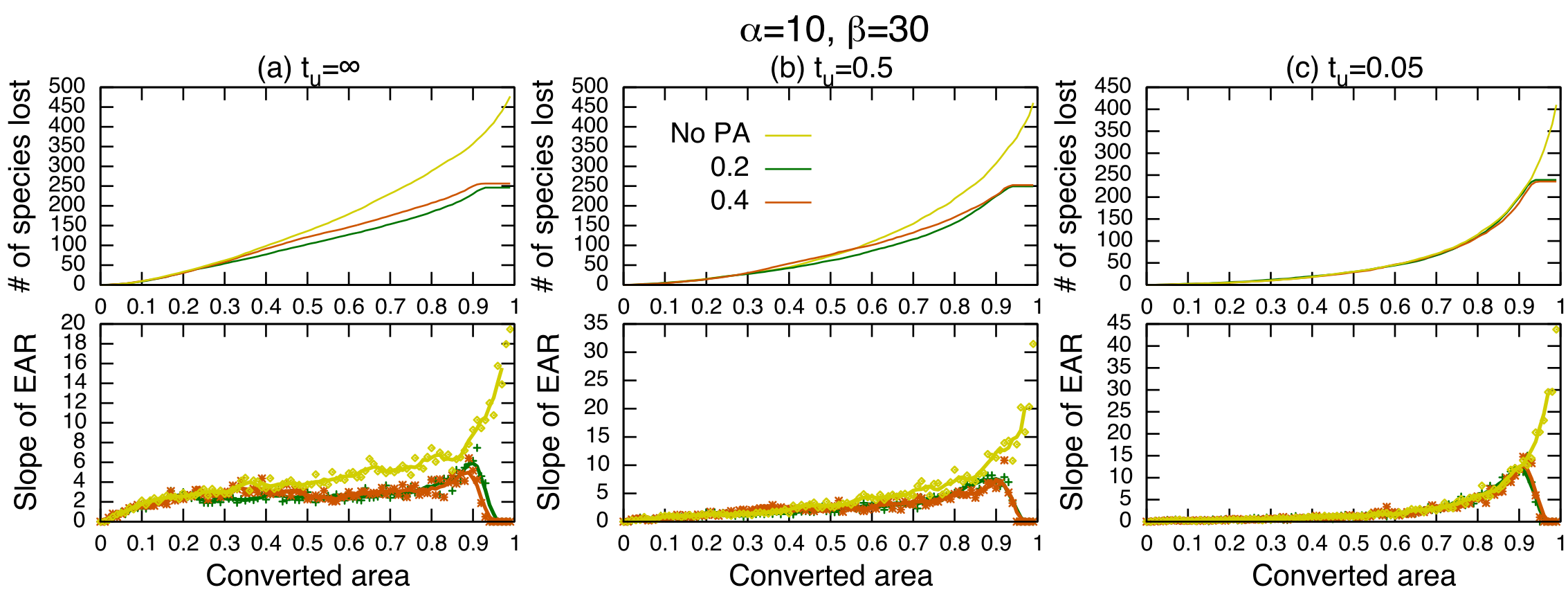

Figure S.4: Effect of protected areas (PAs) on the endemics–area relationships (EARs; top of each panel) and their slope (bottom of each panel) averaged over 100 simulation runs. In the bottom panels, points indicate the estimated slopes, and curves represent five-point moving averages of these values. Protected areas (PAs) introduced 5% in the whole region after the converted area exceeds 20% (green curve) or 40% (red curve). For the comparison, we also plot the no-PAs scenario (yellow curve). Within each panel and simulation run, the scenarios shared the same initial species distributions and richness. The parameter t_u characterizes the frequency with which new land-use occurs, and $t_u = \infty$ indicates that no additional land-use area is formed. Other parameters used (if not specified) are $D_x = D_y = 0.005$.

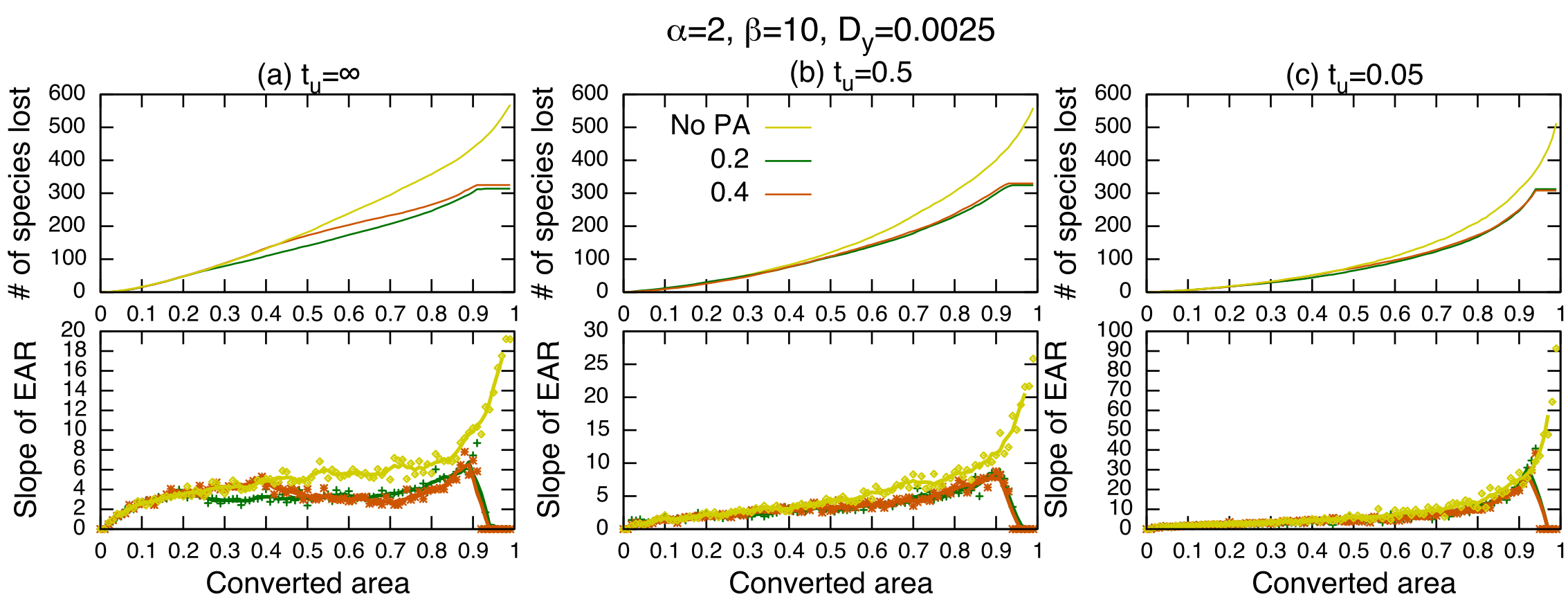

Figure S.5: Effect of protected areas (PAs) on the endemics–area relationships (EARs; top of each panel) and their slope (bottom of each panel) averaged over 100 simulation runs. In the bottom panels, points indicate the estimated slopes, and curves represent five-point moving averages of these values. Non-symmetric rate of land-use expansion was assumed. Protected areas (PAs) introduced 5% in the whole region after the converted area exceeds 20% (green curve) or 40% (red curve). For the comparison, we also plot the no-PA scenario (yellow curve). Within each panel and simulation run, the scenarios shared the same initial species distributions and richness. The parameter t_u characterizes the frequency with which new land-use occurs, and $t_u = \infty$ indicates that no additional land-use area is formed.

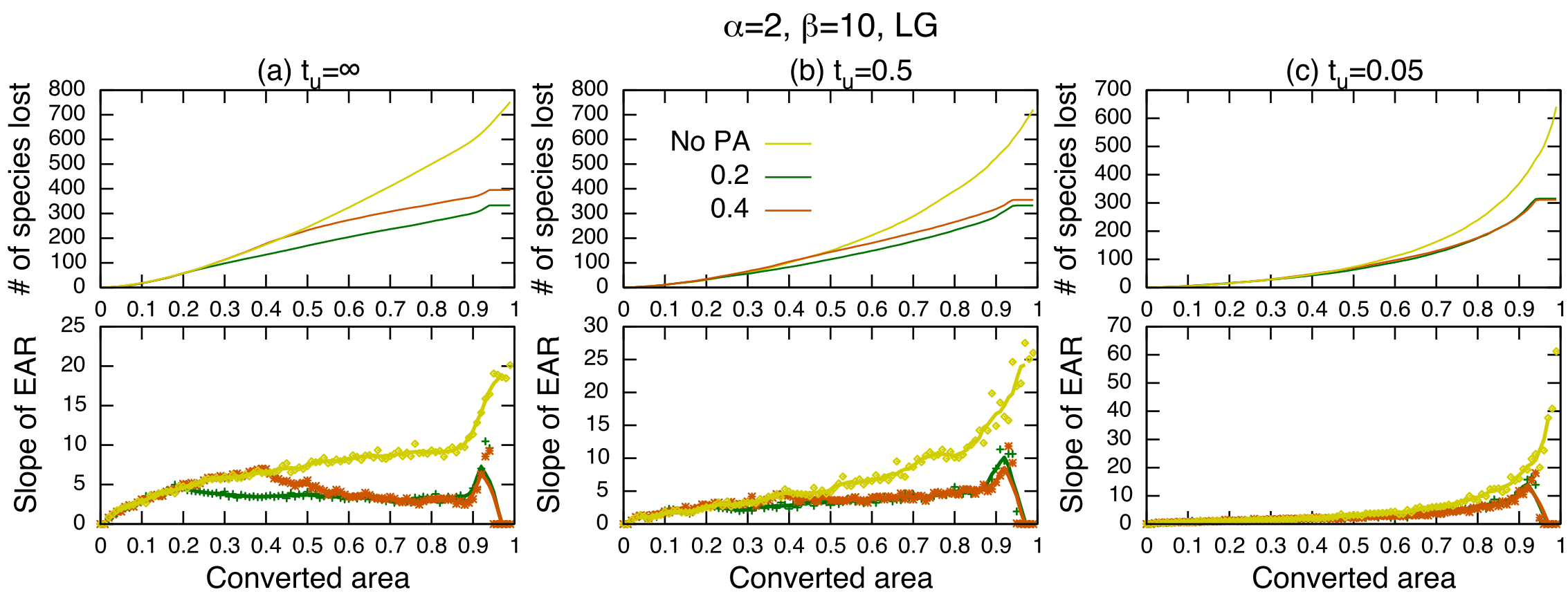

Figure S.6: Effect of protected areas (PAs) on the endemics–area relationships (EARs; top of each panel) and their slope (bottom of each panel) averaged over 100 simulation runs. In the bottom panels, points indicate the estimated slopes, and curves represent five-point moving averages of these values. Latitudinal gradient (LG) was assumed. Protected areas (PAs) introduced 5% in the whole region after the converted area exceeds 20% (green curve) or 40% (red curve). For the comparison, we also plot the no-PA scenario (yellow curve). Within each panel and simulation run, the scenarios shared the same initial species distributions and richness. The parameter t_u characterizes the frequency with which new land-use occurs, and $t_u = \infty$ indicates that no additional land-use area is formed. Other parameters used (if not specified) are $D_x = D_y = 0.005$.

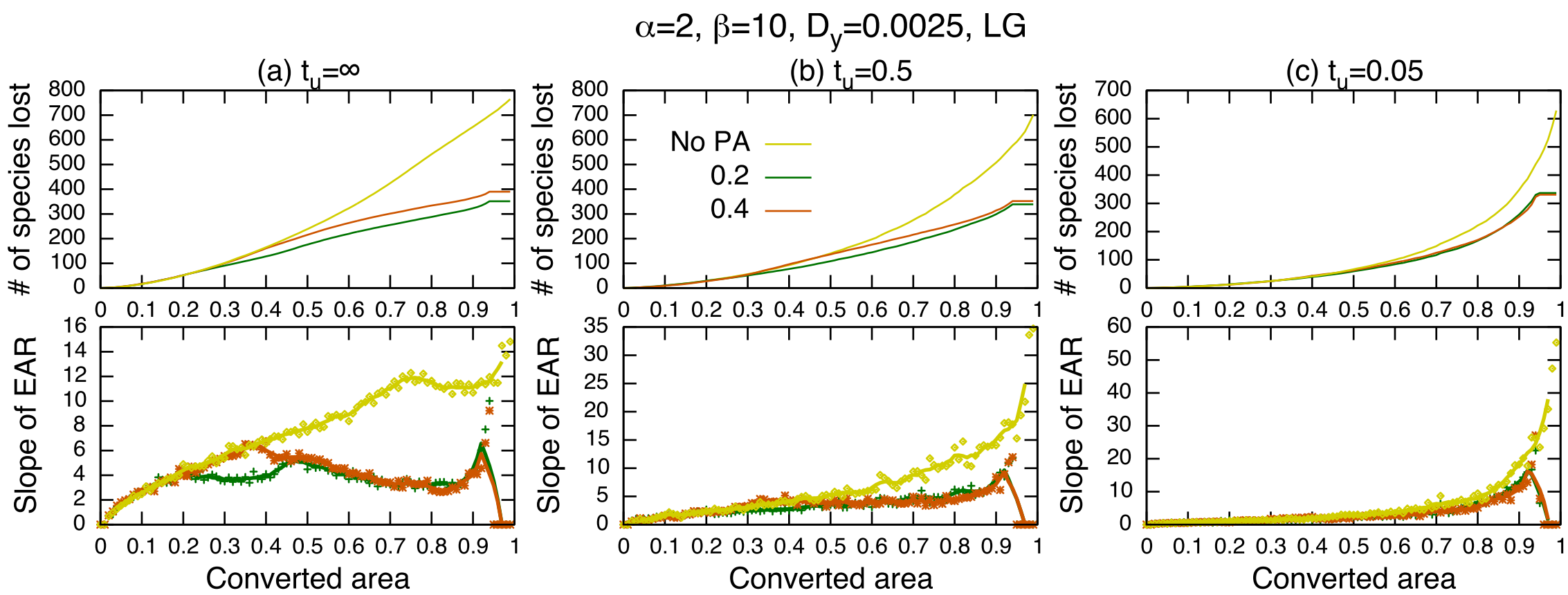

Figure S.7: Effect of protected areas (PAs) on the endemics–area relationships (EARs; top of each panel) and their slope (bottom of each panel) averaged over 100 simulation runs. In the bottom panels, points indicate the estimated slopes, and curves represent five-point moving averages of these values. Latitudinal gradient (LG) and non-symmetric rate of land-use expansion ($D_y = 0.0025$) were assumed. Protected areas (PAs) introduced 5% in the whole region after the converted area exceeds 20% (green curve) or 40% (red curve). The values shown are the averages over 100 simulation runs. For the comparison, we also plot the no-PA scenario (yellow curve). Within each panel and simulation run, the scenarios shared the same initial species distributions and richness. The parameter t_u characterizes the frequency with which new land-use occurs, and $t_u = \infty$ indicates that no additional land-use area is formed.
